# Supplementary material for: Efficacy and Safety of Modified Bismuth Quadruple Therapy for First-Line Helicobacter pylori Eradication: A Systematic Review and Meta-Analysis of Randomized Controlled Trials
Source: Microorganisms. 2025 Feb 26;13(3):519. doi: 10.3390/microorganisms13030519 (PMC11944862; doi:10.3390/microorganisms13030519)
Supplement: Supplementary file 1 [file microorganisms-13-00519-s001.zip › Supplementary Tables-Revised.pdf]

Supplementary Table S1. Search Strategy for PubMed.

NO	Key Words	Search Numbers
#1	"helicobacter"[Title/Abstract]	50260
#2	"pylori"[Title/Abstract]	51279
#3	#1 OR #2	53833
#4	"treatment*"[Title/Abstract] OR "eradication"[Title/Abstract] OR "therap*"[Title/Abstract]	7972141
#5	#3 OR #4	21957
#6	"bismuth"[Title/Abstract]	13573
#7	#5 AND #6	2082

Note: Search Date: 20241231.

Supplementary Table S2. Search Strategy for Embase.

NO	Key Words	Search Numbers
#1	"helicobacter"[Title/Abstract]	67073
#2	"pylori"[Title/Abstract]	72592
#3	#1 OR #2	75919
#4	"treatment*"[Title/Abstract] OR "eradication"[Title/Abstract] OR "therap*"[Title/Abstract]	10984213
#5	#3 OR #4	39607
#6	"bismuth"[Title/Abstract]	14023
#7	#5 AND #6	3195

Note: Search Date: 20241231.

Supplementary Table S3. Search Strategy for Cochrane Library.

NO	Key Words	Search Numbers
#1	"helicobacter"[Title/Abstract]	6364
#2	"pylori"[Title/Abstract]	6924
#3	#1 OR #2	7033
#4	"treatment*"[Title/Abstract] OR "eradication"[Title/Abstract] OR "therap*"[Title/Abstract]	1337440
#5	#3 OR #4	6416
#6	"bismuth"[Title/Abstract]	2186
#7	#5 AND #6	1654

Note: Search Date: 20241231.

Supplementary Table S4. Adverse events associated with *Helicobacter pylori* therapy.

	Total (%)	mBQT (%)	Control (%)	*p*-Value
Mild to moderate events				
Bitter taste	706 (21.2)	303 (19.4)	403 (22.8)	0.016
Abdominal pain	302 (9.1)	155 (9.9)	147 (8.3)	0.109
Nausea or vomiting	739 (22.2)	357 (22.9)	382 (21.6)	0.396
Diarrhea	335 (10.1)	177 (11.3)	158 (9.0)	0.022
Bloating	89 (2.7)	39 (2.5)	50 (2.8)	0.551
Others	1155 (34.7)	530 (34.0)	625 (35.4)	0.378
Severe events	82 (2.5)	32 (2.1)	50 (2.8)	0.146

mBQT, modified bismuth quadruple therapy.
