# Supplementary figures and images for: Efficacy and Safety of Modified Bismuth Quadruple Therapy for First-Line Helicobacter pylori Eradication: A Systematic Review and Meta-Analysis of Randomized Controlled Trials
Source: Microorganisms. 2025 Feb 26;13(3):519. doi: 10.3390/microorganisms13030519 (PMC11944862; doi:10.3390/microorganisms13030519)

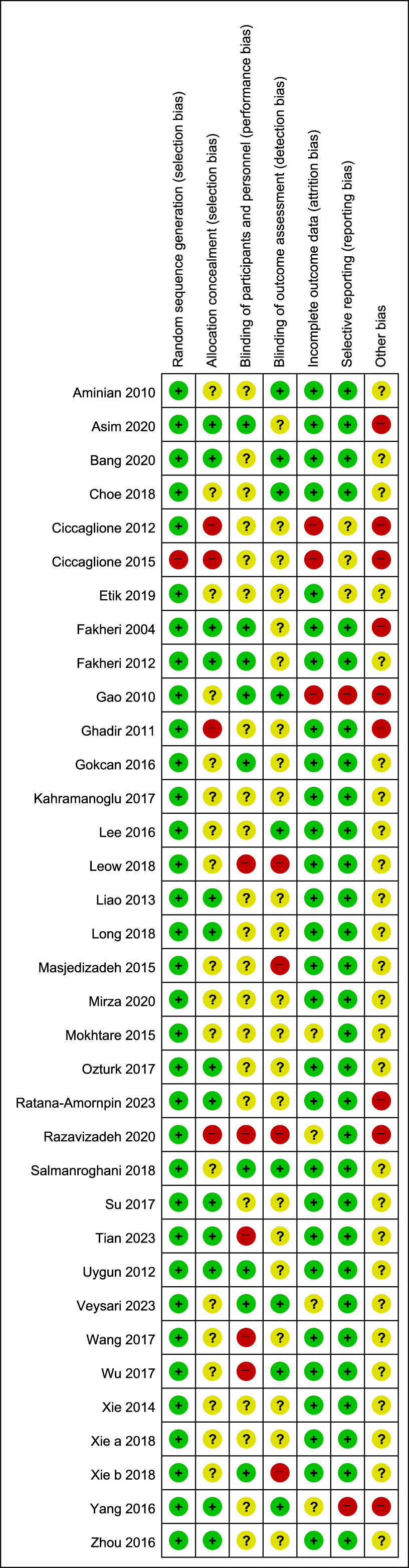

Supplement: Supplementary file 1 [file microorganisms-13-00519-s001.zip › Figure S1.tif]

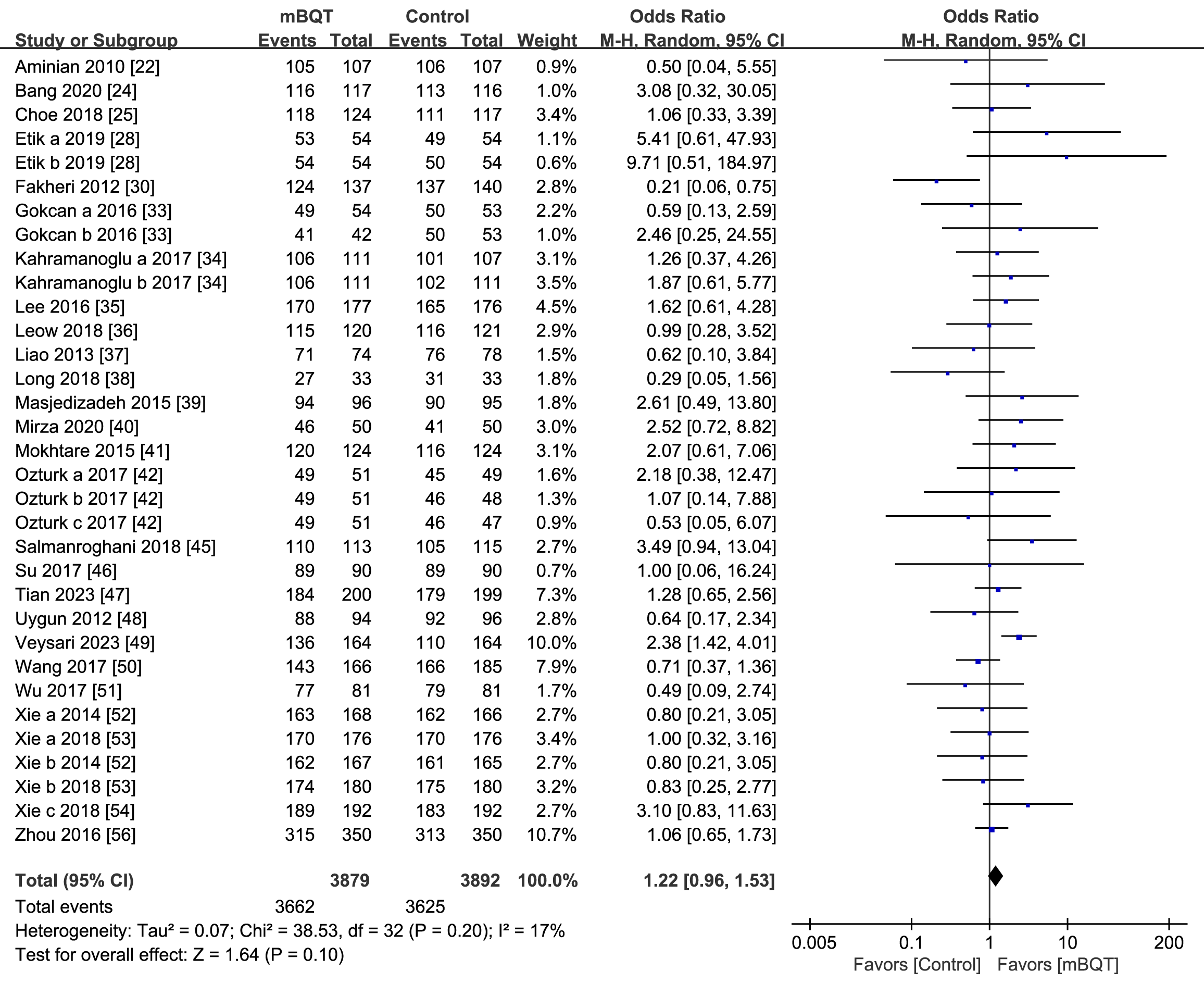

Supplement: Supplementary file 1 [file microorganisms-13-00519-s001.zip › Figure S10.tif]

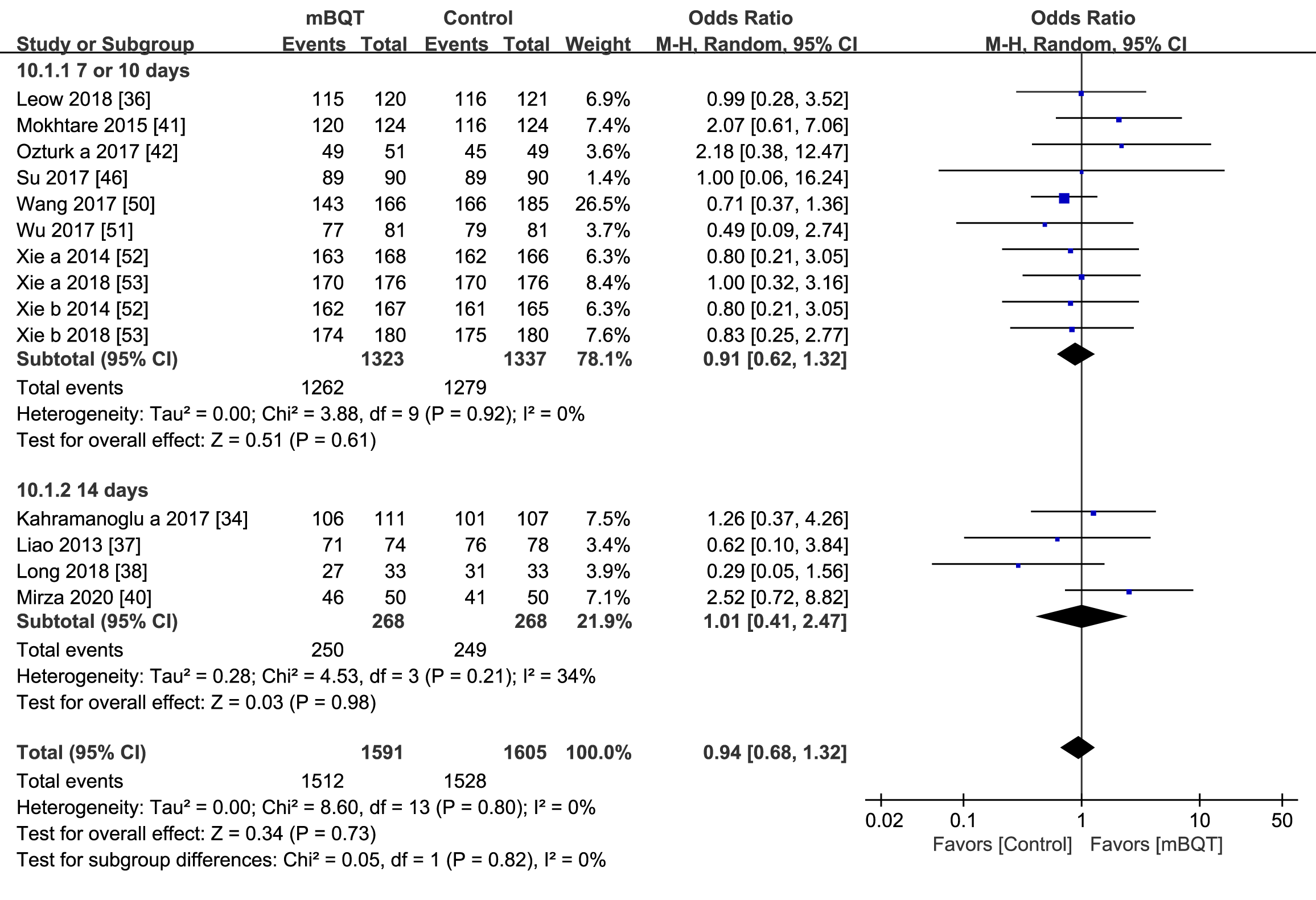

Supplement: Supplementary file 1 [file microorganisms-13-00519-s001.zip › Figure S11.tif]

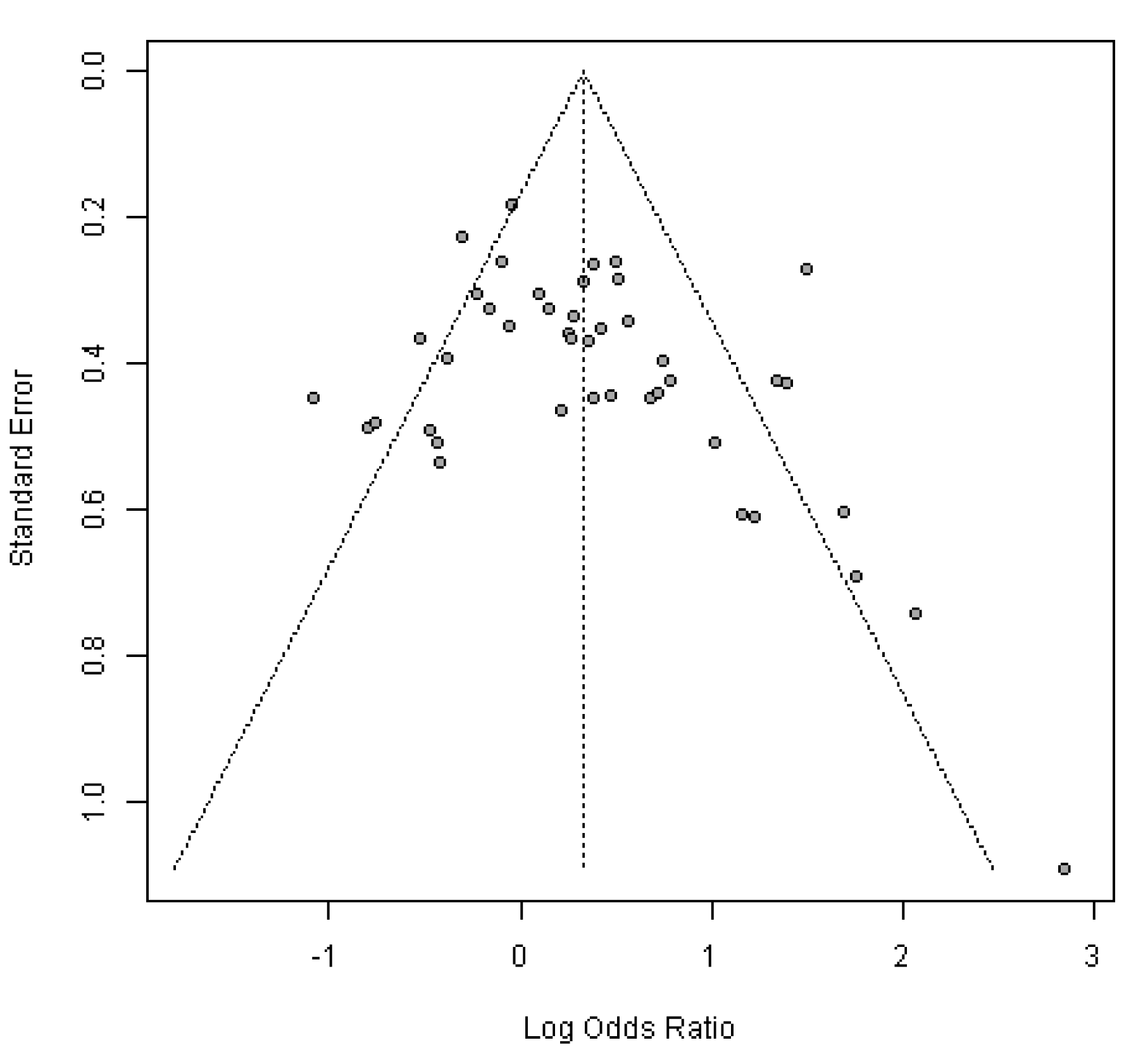

Supplement: Supplementary file 1 [file microorganisms-13-00519-s001.zip › Figure S2.tif]

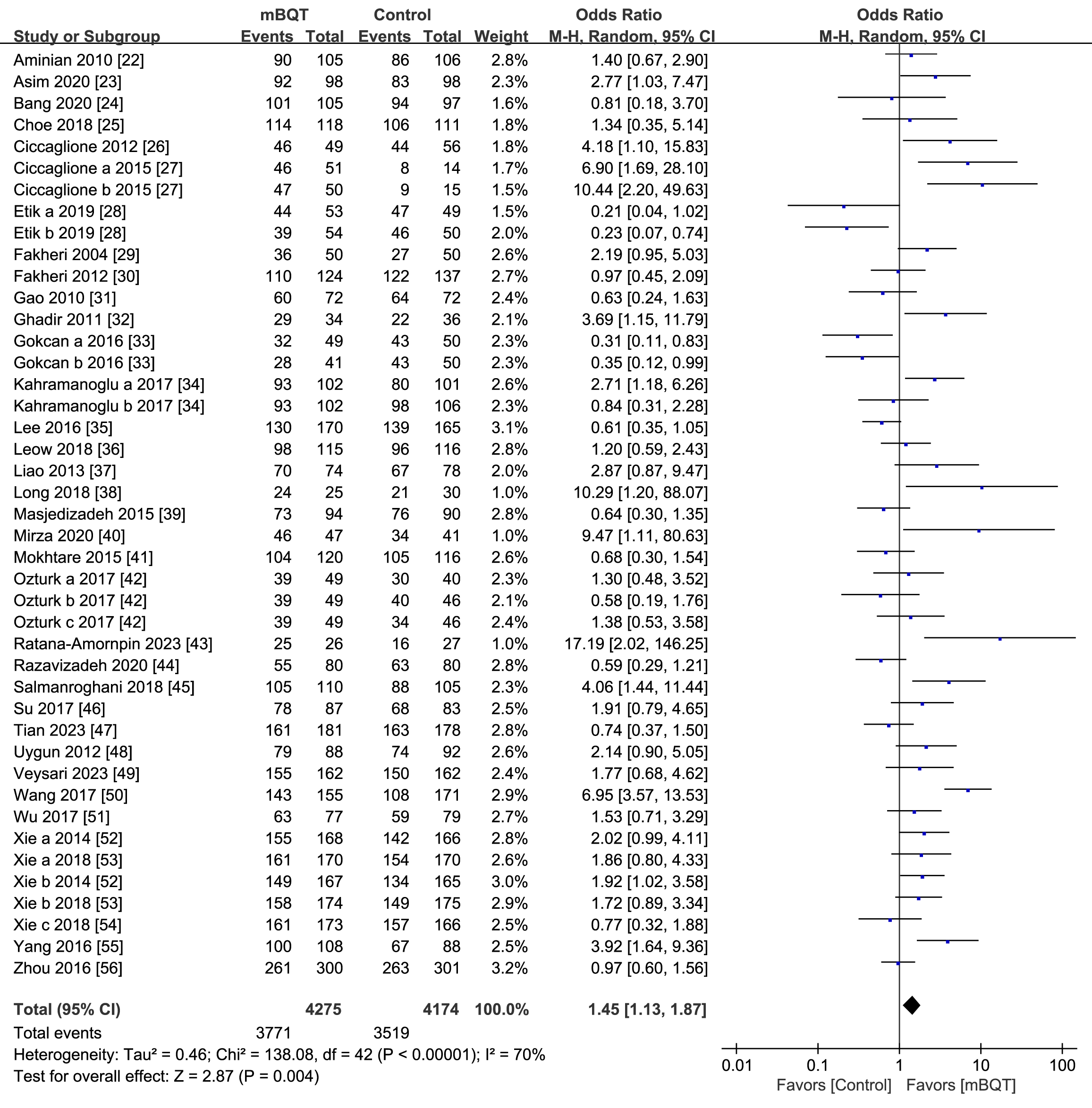

Supplement: Supplementary file 1 [file microorganisms-13-00519-s001.zip › Figure S3.tif]

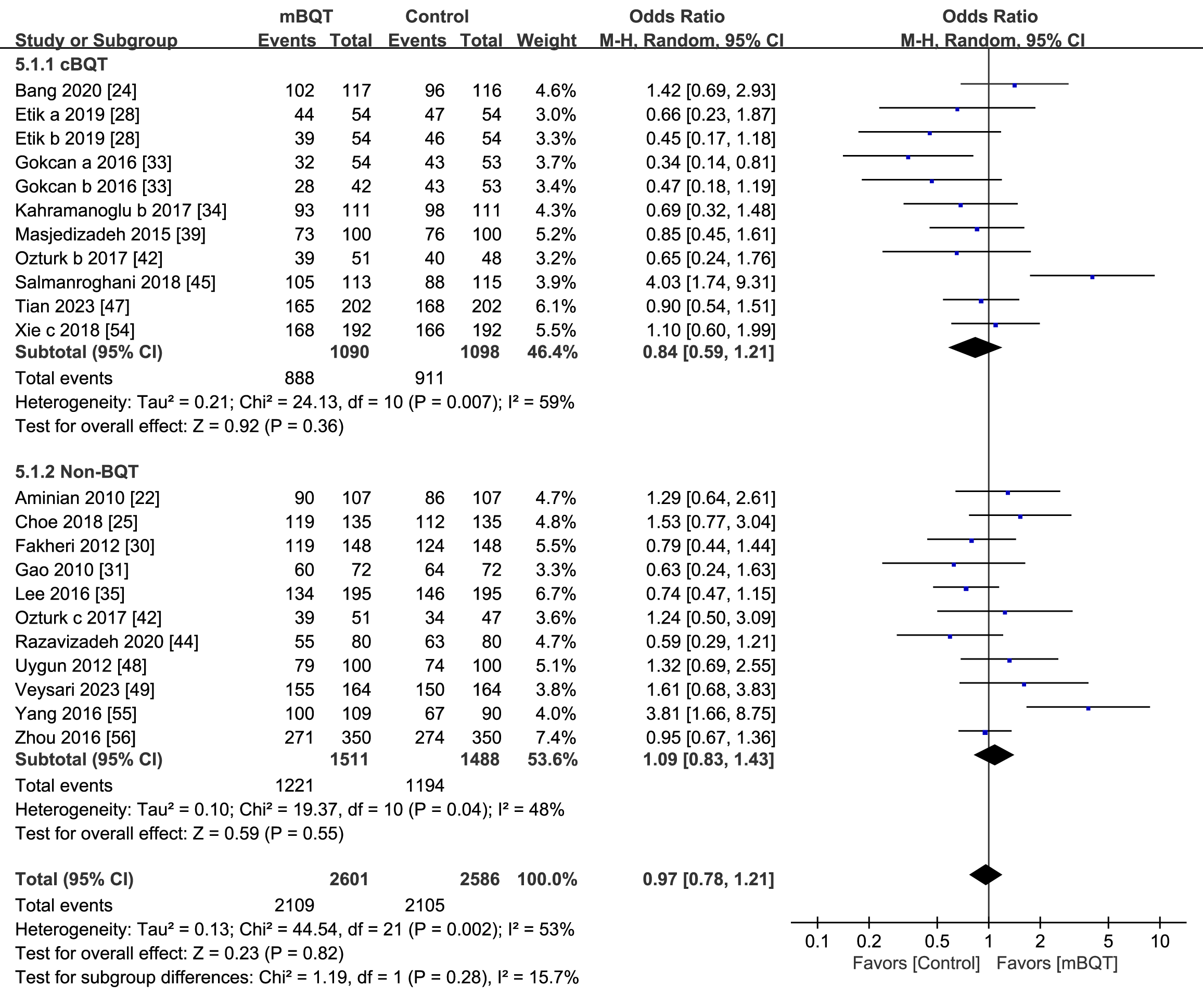

Supplement: Supplementary file 1 [file microorganisms-13-00519-s001.zip › Figure S4.tif]

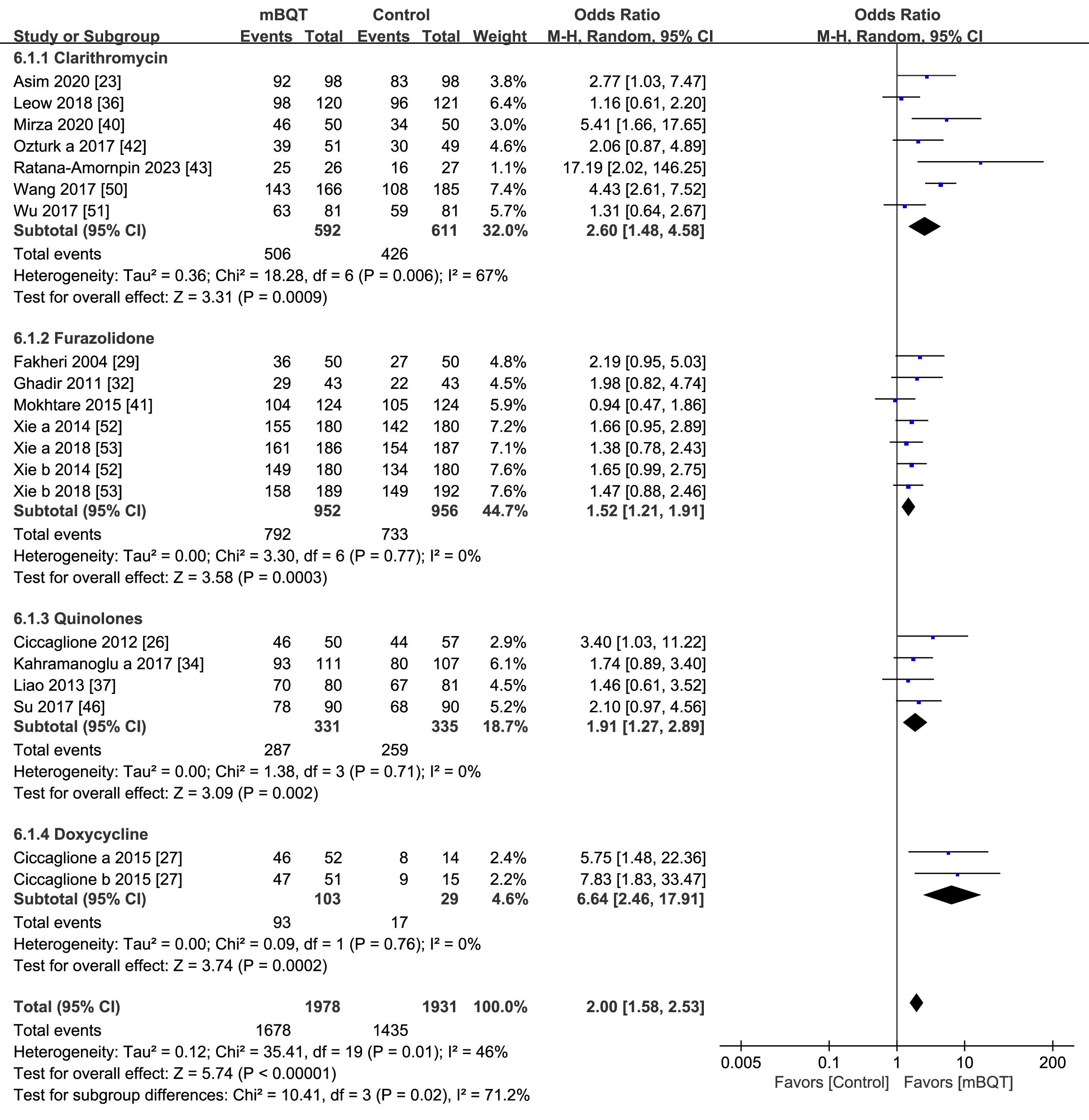

Supplement: Supplementary file 1 [file microorganisms-13-00519-s001.zip › Figure S5.tif]

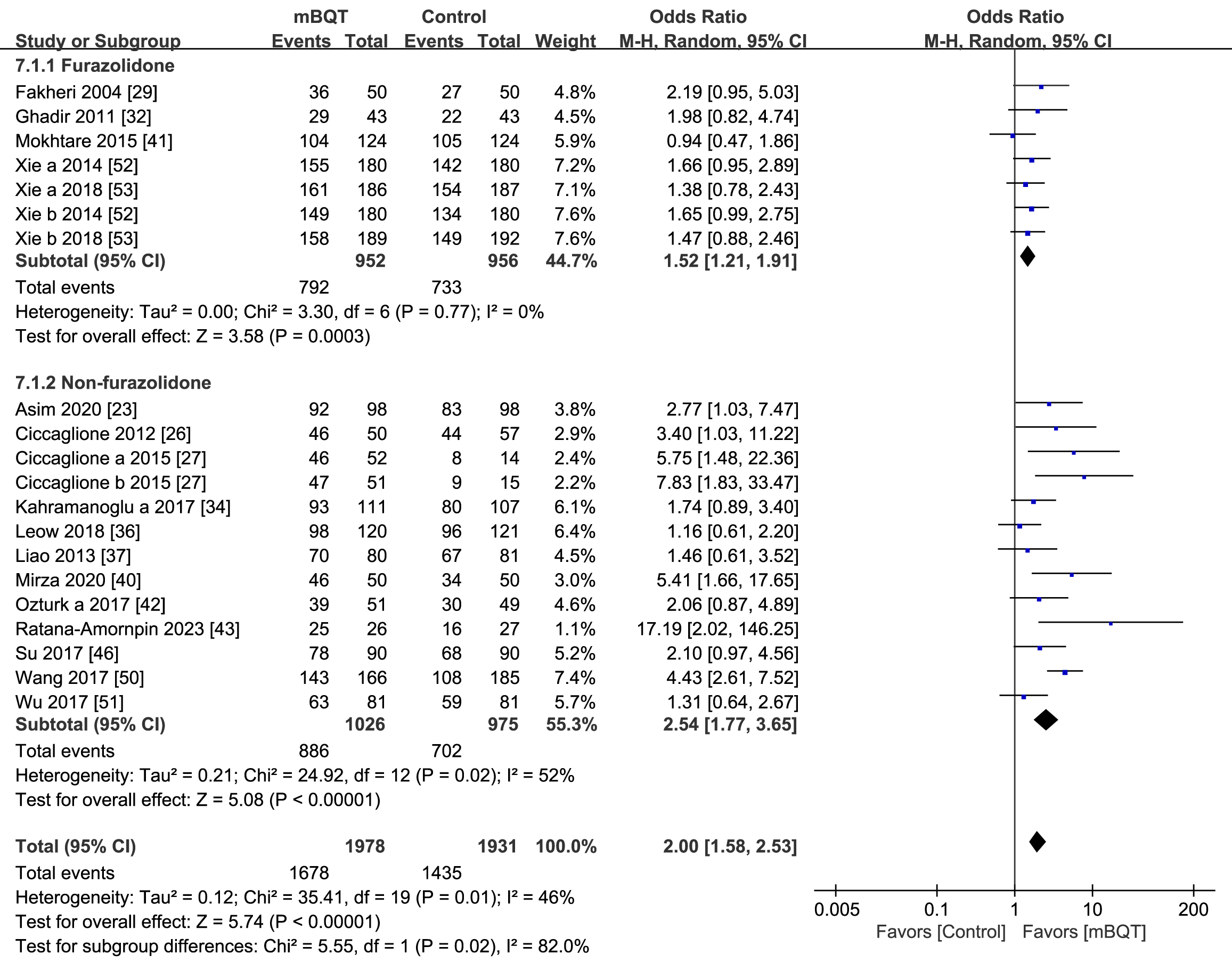

Supplement: Supplementary file 1 [file microorganisms-13-00519-s001.zip › Figure S6.tif]

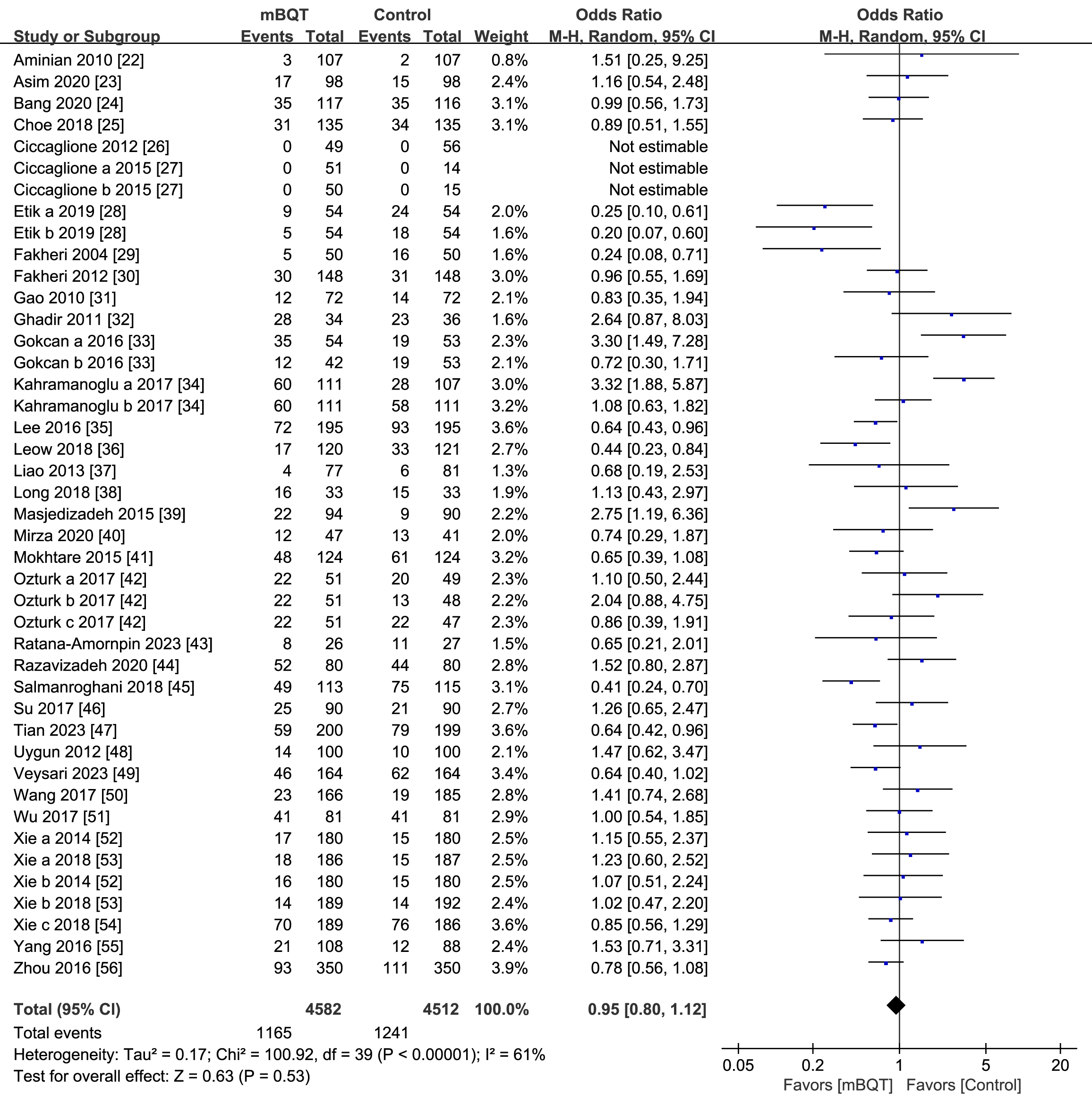

Supplement: Supplementary file 1 [file microorganisms-13-00519-s001.zip › Figure S7.tif]

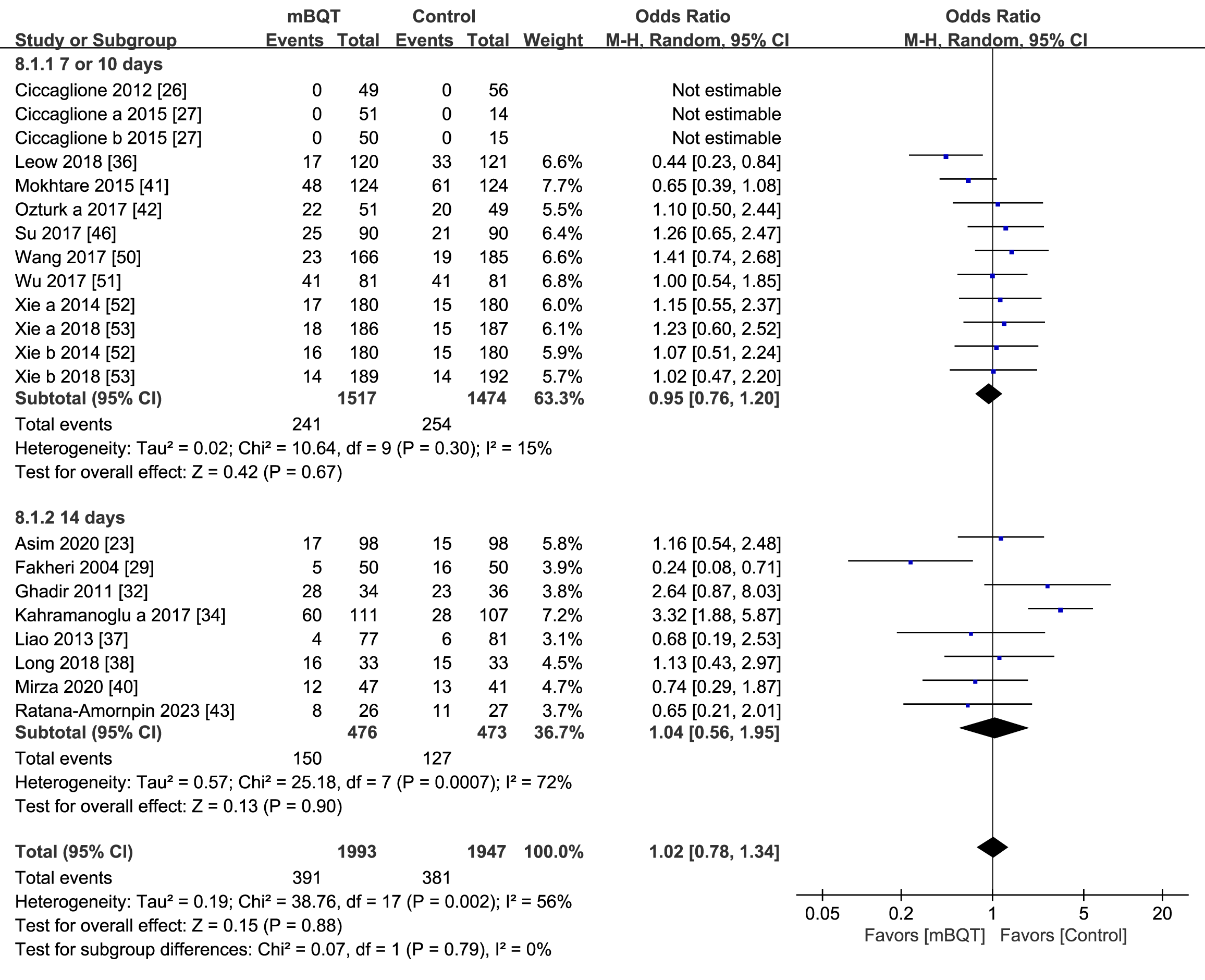

Supplement: Supplementary file 1 [file microorganisms-13-00519-s001.zip › Figure S8.tif]

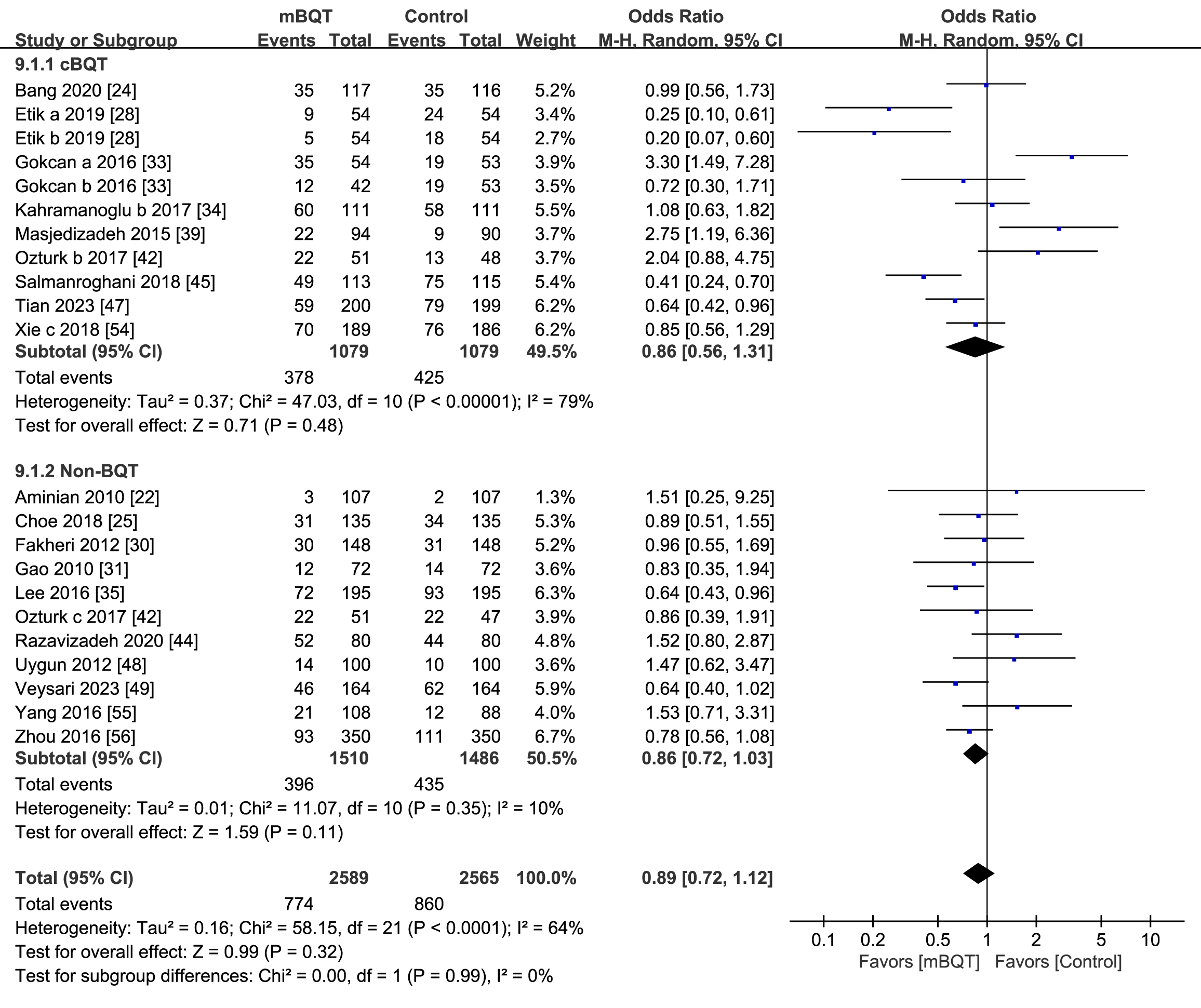

Supplement: Supplementary file 1 [file microorganisms-13-00519-s001.zip › Figure S9.tif]
